# Motionless Polarizing Structured Illumination Microscopy

**DOI:** 10.3390/s21082837

**Published:** 2021-04-17

**Authors:** Hyo Mi Park, Ki-Nam Joo

**Affiliations:** 3D Optical Metrology Laboratory, Department of Photonic Engineering, Chosun University, 309 Pilmun-Daero, Dong-gu, Gwangju 61452, Korea; hyomi0425@gmail.com

**Keywords:** structured illumination microscopy, polarizing illumination pattern, spatial phase-shifting, focus tunable lens

## Abstract

In this investigation, we propose a motionless polarizing structured illumination microscopy as an axially sectioning and reflective-type device to measure the 3D surface profiles of specimens. Based on the spatial phase-shifting technique to obtain the visibility of the illumination pattern. Instead of using a grid, a Wollaston prism is used to generate the light pattern by the stable interference of two beams. As the polarization states of two beams are orthogonal with each other, a polarization pixelated CMOS camera can simultaneously obtain four phase-shifted patterns with the beams after passing through a quarter wave plate based on the spatial phase-shifting technique with polarization. In addition, a focus tunable lens is used to eliminate a mechanical moving part for the axial scanning of the specimen. In the experimental result, a step height sample and a concave mirror were measured with 0.05 µm and 0.2 mm repeatabilities of step height and the radius of curvature, respectively.

## 1. Introduction

Structured illumination microscopy has been widely used for measuring biomedical samples based on fluorescent microscopic techniques [1,2,3,4,5,6,7,8,9,10]. Although widefield fluorescence microscopy is a sensitive method for detecting labeled proteins, it cannot provide a clear image of a sample because of the image blurring caused by the light from out-of-focus regions. To prevent the image blurring, structured illumination microscopy uses a structured illumination pattern to computationally eliminate the out-of-focus light and enhances the fluorescent image quality of the sample. Typically, structured illumination microscopy has been developed as two categories: lateral resolution enhancement, so-called super-resolution structured illumination microscopy (SR-SIM) [1,2,3,4,5]; and 3D optical sectioning structured illumination microscopy (SIM) [6,7,8,9,10]. Conventional optical microscopy only has a limited lateral resolution due to the Abbe diffraction limit, and also lacks the ability of 3D imaging. However, SR-SIM enhances lateral resolution by collecting several patterned images and analyzing them in reciprocal space, such as in the Fourier domain [1,2,3,4,5]. On the other hand, SIM can provide 3D imaging of the sample by detecting the visibility (modulation depth) of the illumination pattern [6,7,8,9,10,11,12]. 

Beyond the biomedical imaging, SIM can also measure the 3D surface profiles of various specimens, such as semiconductor and display products [11,12] without fluorescent imaging. By using a sinusoidal amplitude grating, such as a Ronchi grating, and a grid, in this case, the spatial light pattern can be imaged to the surface of the specimen and obtained by an image sensor to calculate its visibility. Similar to confocal scanning microscopy (CSM), the specimen is axially scanned, and SIM can detect the position of the maximum visibility to find out the surface height of the specimen. SIM does not need expensive and specially designed optical components, i.e., a Nipkow disk and a micro-lens array, to obtain 3D surface profiles, and it is a good and efficient alternative to replace CSM.

The visibility of the sinusoidal pattern in SIM can be extracted by the phase-shifting technique [13] or the Fourier method [14,15]. Although the linear [13] and rotational motion of a grid can be simply implemented for shifting the light pattern, it needs a mechanical moving part, which leads to the measurement errors of SIM caused by phase-shifting errors and mechanical vibrations. In order to overcome this limitation, the grid can be replaced with a spatial light modulator (SLM) [16,17] or a digital micromirror device (DMD) [18] to generate the pattern. However, the phase-shifting of the pattern in SIM is still operated in serial, which means several phase-shifted images should be obtained at an axial position of the specimen [13]. This temporal phase-shifting technique is a fundamentally limiting factor of the long measurement time of SIM.

On the other hand, a spatial phase-shifting technique has been reported in various optical interferometers [19,20,21,22] to obtain the phase at once, as opposed to the temporal phase-shifting technique. Based on the polarization states of a reference and a measurement beam, four phase-shifted interferograms can be simultaneously captured, and do not need the temporal acquisition of interferograms. Recently, a polarization pixelated CMOS camera (PCMOS) has been commercially available and used in spatial phase-shifting interferometers [20,21,22]. However, in order to apply the spatial phase-shifting technique, the polarization status of one beam should be orthogonal to that of the other beam, and several polarizing optical components should be used in the system. 

In this investigation, we propose a novel type of SIM, namely motionless polarizing SIM (MP-SIM), which adopts the spatial phase-shifting technique to obtain the visibility of the pattern as an axially sectioning and reflective-type device. Instead of using a grid, MP-SIM uses a Wollaston prism to generate the light pattern by the stable interference of two beams, as the polarization states of two beams are orthogonal with each other; moreover, the spatial phase-shifting can be simply implemented, and four phase-shifted patterns are captured by a PCMOS at once. Therefore, MP-SIM can obtain the visibility of the pattern immediately at an axial position of the specimen. Additionally, MP-SIM uses a focus tunable lens to eliminate a mechanical moving part for axial scanning of the specimen used in typical SIM. 

The proposed MP-SIM uses a polarizing illumination pattern to apply the spatial phase-shifting technique for obtaining the visibility of the signal opposed to typical SIM. All of the relevant research works have used the typical illumination pattern by a grid, DMD, or SLM, and the visibility was able to be obtained in the temporal way. However, the proposed system can obtain the visibility at once. The proposed system approaches the system modification with a completely different way, not tried previously. Currently, recent research work relevant to SIM has been implemented as two categories: biomedical applications [7,8,9,10] and system modifications [23,24,25,26,27]. For the system modification of SIM to enhance the measurement sensitivity and speed, they used two cameras for differential operation [23], fast temporal phase-shifting [24], deep-learning [25], iterative algorithm [26], and HiLo technique [27]. MP-SIM in this investigation is also involved in the system modification of SIM with a novel measuring principle.

## 2. Methods

### 2.1. Principle of Structured Illumination Microscopy (SIM)

**Figure 1:**
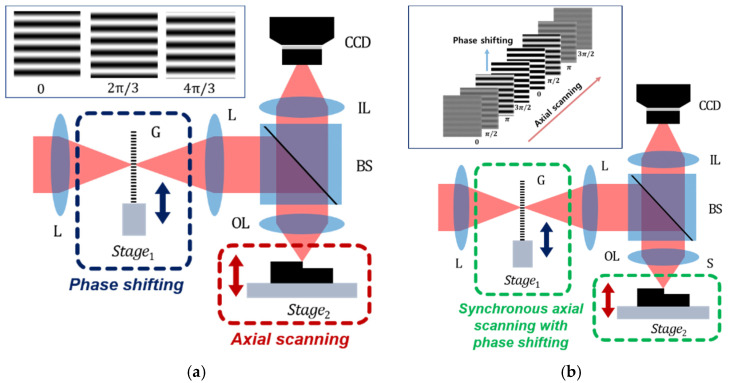
Optical configurations and operating principles of (**a**) typical structured illumination microscopy (SIM) and (**b**) continuously scanning structured illumination microscopy (CSSIM); L, lens; G, grid; BS, beam splitter; OL, objective; S, specimen; IL, imaging lens; CCD, charge coupled device.

Figure 1a shows the optical configuration of a typical SIM. A sinusoidal amplitude grating is used for generating the spatial light pattern, and the patterned beam is incident to the specimen. In this case, the surfaces of the grating, the specimen, and the imaging plane of the CCD camera are optically conjugated, which means that the pattern can clearly appear on the imaging plane when the specimen is located at the best focus of an objective. In order to calculate the visibility of the pattern, the phase-shifting technique is applied, and the grid is laterally shifted with a 2π/3 phase step [13]. Then, the visibility of the pattern (*V*) can be described as

(1) $$V={\left[{\left(I_{1}-I_{2}\right)}^{2}+{\left(I_{1}-I_{3}\right)}^{2}+{\left(I_{2}-I_{3}\right)}^{2}\right]}^{1/2}$$

where *I*_1_, *I*_2_, and *I*_3_ are the intensities with the phase shifts of 0, 2π/3, and 4π/3, respectively. It is noted that the lateral coordinate (x,y) is omitted in Equation (1) for simplicity. As the specimen is axially moved, SIM can obtain the visibility curves for all pixels of the camera and detect the positions of the maximum visibility points to reconstruct the 3D surface profile of the specimen. However, the axial motion of the specimen is not synchronized with the lateral motion of the grid in SIM, and is operated in the so-called ‘stop-and-go’ method for extracting the visibility of the pattern at each axial position of the specimen. 

**Figure 2:**
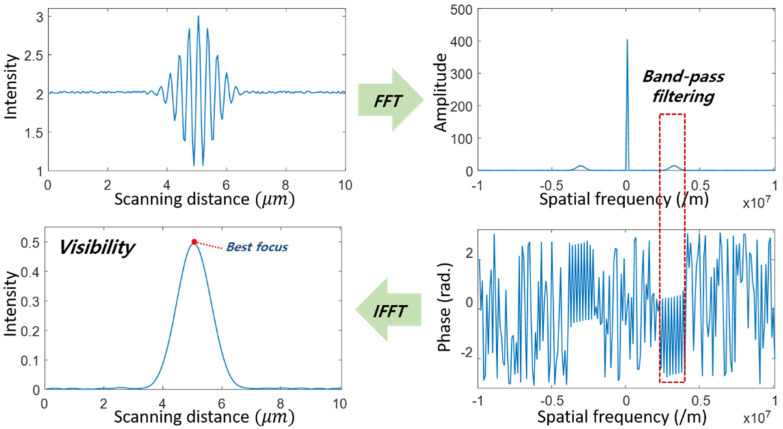
Measurement algorithm of CSSIM based on the Fourier transformation and the inverse Fourier transformation.

A continuously scanning SIM (CSSIM) [14] uses the synchronization between the lateral phase-shifting of the grid and the axial motion of the specimen to reduce the measurement time caused by the ‘stop-and-go’ method of SIM. In CSSIM, the visibility curve of each pixel corresponding to the surface height can be extracted from the axial intensity variation by a Fourier transformation (FT) and an inverse Fourier transformation (IFT), as shown in Figure 2. A simple mathematical model of the axial intensity signal I(z) at a single pixel in CSSIM can be expressed with the peak position (h) and axial scanning position (z) [15] as

(2) $$I\left(z\right)=I_{0}+I_{C}\left(z-h\right)\times \cos\left(2\pi f_{S}z+\varphi_{0}\right)$$

where $I_{0}$ is a nominal intensity. $f_{S}$ and $\varphi_{0}$ mean the modulation frequency and the initial phase caused by the phase-shifting of the sinusoidal pattern, respectively. In order to obtain the visibility curve $I_{C}\left(z-h\right)$ from I(z), an FT is applied to Equation (2), and the result is inversely Fourier transformed after being band-pass filtered to select only a positive term in the Fourier domain. Then, the filtered intensity ($I_{F}$) can be derived as

(3) $$I_{F}=\frac{1}{2}I_{C}\left(z-h\right)\times exp\left[j\left(2\pi f_{S}z+\varphi_{0}\right)\right]$$

where $I_{C}\left(z-h\right)$ can be calculated by the absolute value of $I_{F}$. The peak position of $I_{C}\left(z-h\right)$ is then obtained by a simple mathematical technique such as a center of gravity. As the phase-shifting is synchronized with the axial motion, this intensity variation is very similar to the correlogram of low-coherence scanning interferometry (LCSI), and its envelope peak corresponding to the maximum visibility of the pattern can be detected by the various algorithms used in LCSI [28,29]. However, the visibility of the signal in CSSIM can be only extracted after the whole acquisition of the images, and cannot be obtained during the measurement procedure.

Moreover, the pattern should still be phase-shifted in the previous works on SIM and CSSIM, and can cause measurement errors. If not exactly phase-shifted because the grid is not precisely moved, the phase-shifting errors deteriorate the visibility of the pattern, and the reconstructed surface profile is distorted. In addition, the serial acquisition of phase-shifted images fundamentally takes a long time, which induces measurement errors caused by environmental factors such as vibrations.

### 2.2. Principle of Motionless Polarizing Structured Illumination Microscopy (MP-SIM)

**Figure 3:**
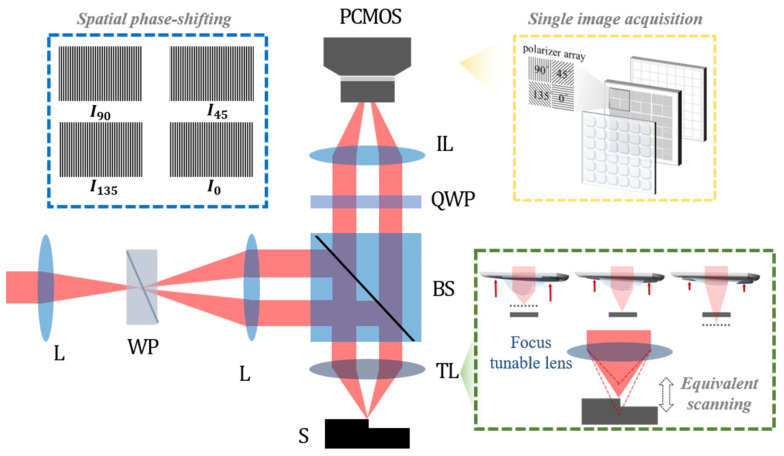
Optical configuration of the motionless polarizing structured illumination microscopy (MP-SIM); L, lens; WP, Wollaston prism; BS, beam splitter; TL, focus tunable lens; S, specimen; QWP, 45° rotated quarter-wave plate; IL, imaging lens; PCMOS, polarization pixelated camera.

In order to avoid mechanical motions of SIM, our system, motionless polarizing structured illumination microscopy (MP-SIM), adopts a new principle of generating a sinusoidal light pattern using polarizing optical components and a polarization pixelated camera (PCMOS), as shown in Figure 3. 

The light beam from the optical source is split into two orthogonally polarized beams with each other by a Wollaston prism (WP), and they are overlapped on the surface of the specimen. Due to the orthogonal polarization states, two beams cannot generate the interference pattern, but it can be detected by the PCMOS, where four kinds of polarizer arrays with different transmission directions are attached in front of the imaging sensors. Moreover, the PCMOS can capture 4 phase-shifted interference patterns at once after the two linearly polarized beams pass through a 45° rotated quarter-wave plate (QWP). Based on this spatial phase-shifting technique, the visibility of the pattern can be directly obtained. MP-SIM also uses a focus tunable lens (TL) to eliminate the axial motion of the specimen.

In MP-SIM, all kinds of mechanical motions are eliminated, and the visibility can be obtained without temporal operations. It is the reason why MP-SIM is robust for environmental vibrations. In MP-SIM, alignment errors can affect the robustness of the system because of the alignment of the Wollaston prism, but the effect can be significantly reduced after being well pre-aligned. Even though SIM and CSSIM are easy to implement, MP-SIM has the new features such as polarizing illumination pattern and spatial phase-shifting.

#### 2.2.1. Polarizing Structured Illumination

**Figure 4:**
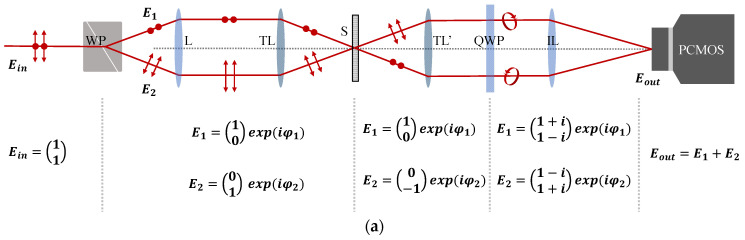

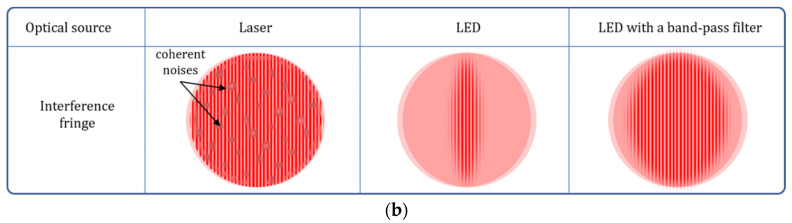
(**a**) Beam propagation in MP-SIM with the polarization state and (**b**) interference fringe corresponding to the coherence of each light source. It is noted that the prime symbol means ‘reversely directed’.

Figure 4a shows the beam propagation in MP-SIM considering polarization states. As opposed to typical SIM, which generates structured illumination by imaging a grid, MP-SIM uses the interference between two beams split by the WP. The propagation directions of two beams (*E*_1_ and *E*_2_) are symmetrically deviated from the original one with the aid of the operation principle of the WP, and they generate the interference pattern, which can be highly modulated along the vertical direction for the structure illumination of MP-SIM. This polarizing structured illumination has two kinds of different features compared to the typical one. One is the pattern that can be only shown in the PCMOS, which uses linear polarizers. The other is the pattern that can be affected by the temporal and spatial coherence of the light source, as shown in Figure 4b. When a laser light that has a monochromaticity and directionality is used, the interference fringe can clearly appear, but some coherent noises such as diffraction and speckle pattern are contained in the image. Moreover, the interference does not disappear even when the specimen is out of focus because of the high coherence of the light. When a lamp or LED light is used, on the contrary, the coherent noises can be removed by the low spatial coherence. However, this broadband light source only generates a localized interference pattern because of its low coherence. In this investigation, therefore, we used an LED with a narrow band-pass filter to restrict the coherent noises as well as to mitigate the localized interference pattern for the structured illumination of MP-SIM.

#### 2.2.2. Spatial Phase-Shifting Technique for Calculating the Visibility of the Pattern

After passing through the QWP, as shown in Figure 4a, two linearly polarized beams are converted into a circularly polarized beam with different rotations, i.e., one beam is right-hand circularly polarized and the other is left-hand circularly polarized. These two beams can generate four phase-shifted interference patterns in the PCMOS based on the transmission axes of a polarizer array with 0°, 45°, 90°, and 135° as [30].

$$I_{0}={\left|E_{1,0}+E_{2,0}\right|}^{2}=A\left(1+\gamma sin\varphi \right)$$

$$I_{45}={\left|E_{1,45}+E_{2,45}\right|}^{2}=A\left(1+\gamma cos\varphi \right)$$

$$I_{90}={\left|E_{1,90}+E_{2,90}\right|}^{2}=A\left(1-\gamma sin\varphi \right)$$

(4) $$I_{135}={\left|E_{1,135}+E_{2,135}\right|}^{2}=A\left(1-\gamma cos\varphi \right)$$

where *I*_0_, *I*_45_, *I*_90_, and *I*_135_ are the intensity detected by 4 different pixel sets of the PCMOS, respectively, and *A* is the mean intensity of the interference fringe. *φ* means the phase difference between two beams (*E*_1_ and *E*_2_), and *γ* indicates the visibility of the interference fringes (the modulation depth of MP-SIM). As the interference fringes in Equation (4) are shifted as 90° successively, they can induce *γ* as

(5) $$\gamma =\frac{2\sqrt{{\left(I_{0}-I_{90}\right)}^{2}+{\left(I_{45}-I_{135}\right)}^{2}}}{I_{0}+I_{90}+I_{45}+I_{135}}$$

The use of PCMOS enables the phase-shifting spatially, and MP-SIM can obtain *γ* at each axial position of the specimen at the moment. Therefore, MP-SIM can avoid the temporal scanning of the pattern, which invokes mechanical vibrations and measurement errors by the scanning motions. 

#### 2.2.3. Axial Scanning by a Focus Tunable Lens

**Figure 5:**
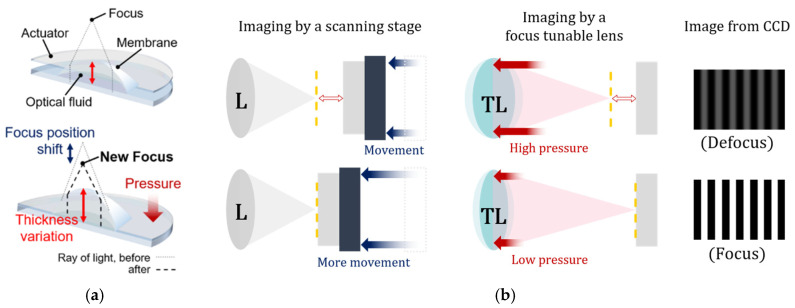
(**a**) Operation principle of a focus tunable lens (TL) and (**b**) axial scanning by using a TL compared with the mechanical motion.

The axial scanning of the specimen in typical SIM is replaced with the adjustment of focal length of the TL in MP-SIM. A focus tunable lens consists of a membrane, optical fluid, and an electric coil as shown in Figure 5a. When the current is applied to the coil, the pressure corresponding to the current is induced in the fluid, which varies the shape of the membrane, especially the center thickness of the lens. In this case, the focus adjustment of the TL is as same as the axial scanning of the specimen, as shown in Figure 5b.

## 3. Results

### 3.1. Pattern Generation by a Wollaston Prism

**Figure 6:**
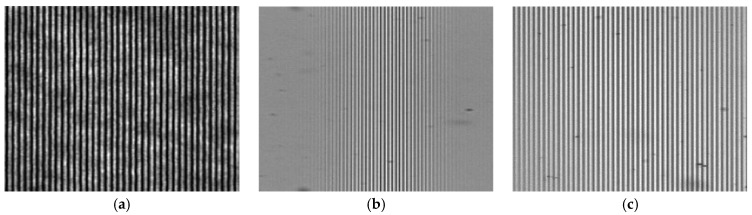
Illumination patterns generated by the WP using (**a**) a He-Ne laser, (**b**) white LED and (**c**) white LED with 1 nm band-pass filter.

Before measuring the 3D surface profiles of specimens, a polarizing structured illumination pattern was generated by the WP with three kinds of optical sources, a He–Ne laser and a white LED with and without a narrow-band-pass filter. The illumination pattern was projected to a plane mirror and imaged to the PCMOS. As the WP, a commercialized Wollaston prism (WPQ10, Thorlabs) with 1° beam separation, was used, and the patterns were observed by the PCMOS, a commercialized polarization pixelated camera (PHX050S-PC, Lucid) with 2448 × 2048 pixels. Figure 6 shows the generated patterns of the 0° polarized image of the PCMOS using three optical sources when the mirror was located at the best focus of the TL.

As shown in Figure 6a, the illumination pattern using a He–Ne laser had the highest contrast, but the image contained the coherent noises like stains. Additionally, the pattern kept having high contrast in spite of the axial scanning of the mirror. On the other hand, the pattern using a white LED was localized, and only appeared near the central area of the image as shown in Figure 6b because of the low coherence. These two patterns were not well matched to the conditions of the illumination pattern in SIM because of severely high and low coherence characteristics. To mitigate this, a narrow band-pass filter with 1 nm bandwidth was used with the white LED in this investigation, which means the light has intermediately low temporal coherence to extend the pattern area and low spatial coherence to eliminate the coherent noises. As the result, the pattern clearly appeared at the whole area of the imaging plane without significant coherent noises as shown in Figure 6c.

### 3.2. 3D Surface Profile Measurements by MP-SIM

Based on Figure 3, MP-SIM was constructed as a 5x microscope system in this investigation. The optical source part was composed of a white LED, a condenser, a 1 nm band-pass filter, and the WP to generate the proper polarizing illumination pattern, as mentioned in Section 3.1. 

**Figure 7:**
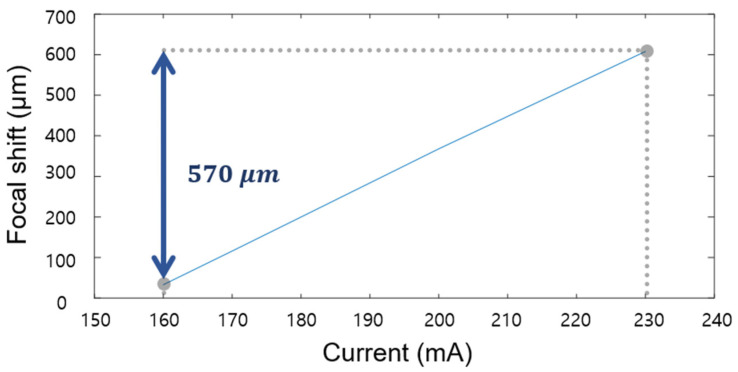
Calibration result of the focal shift and the current of the TL.

The TL was located between a 5× objective and a specimen to adjust the working distance. To find out the relationship between the current and the focal length shift of the TL, a micro-stepping motorized stage was used, and the image stack of the polarizing illumination pattern was captured by the PCMOS camera as the current of the TL increased. The visibilities of the pattern were calculated along the 100 nm stepping motion of the stage at each current value of the TL, and the peak position of the visibility curve was determined. The current of the TL was adjusted from 160 mA to 230 mA with 10 mA step, and the total focal shift indicated that the stage movement was 570 µm. As a result, it was confirmed that the current values had a linear relationship with the focal length shifts, as shown in Figure 7, with a conversion factor of 8.14 µm/mA.

**Figure 8:**
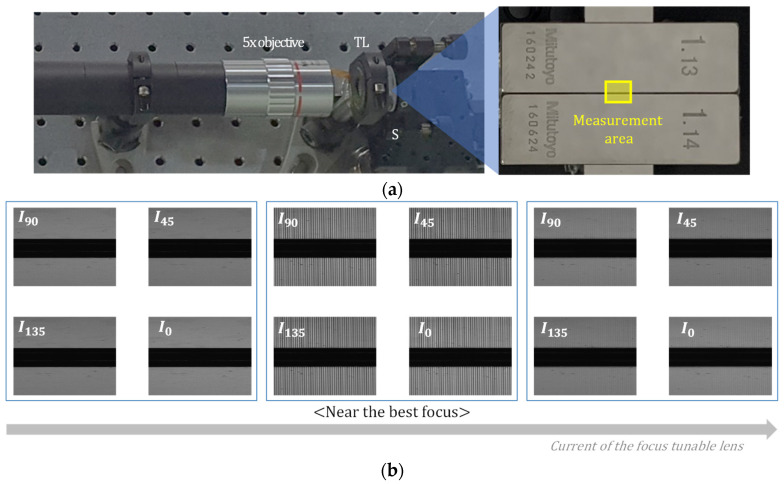
(**a**) Step height sample which consists of two gauge blocks and (**b**) an illumination pattern as the current of the focus tunable lens increased.

**Figure 9:**
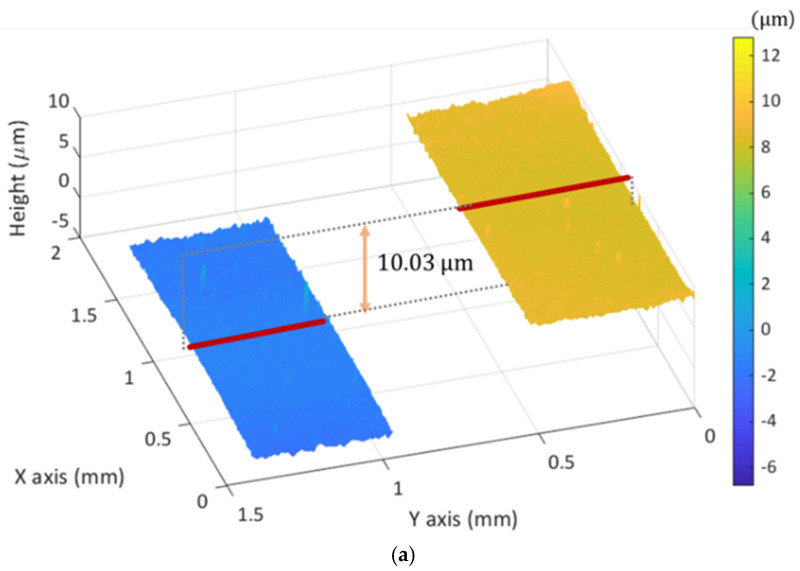

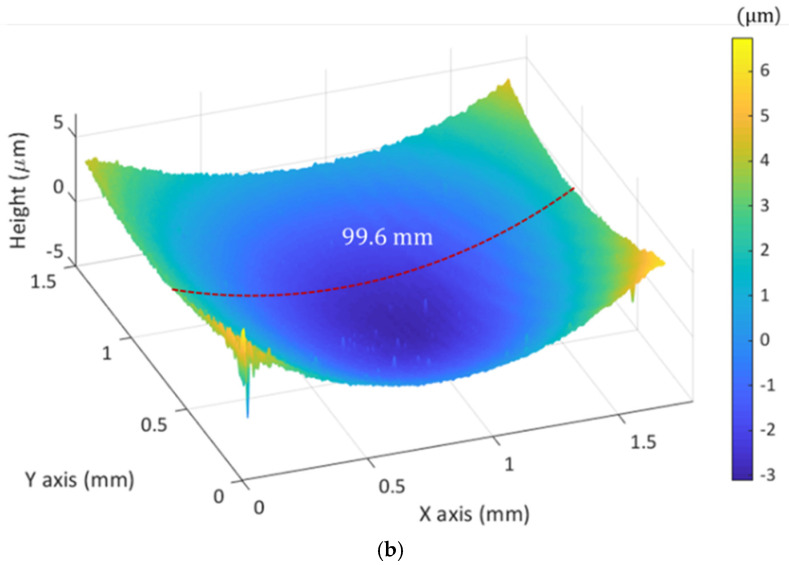
Reconstructed 3D surface profile of (**a**) the step height sample with a 10 μm height step and (**b**) the concave mirror with a 100 mm radius of curvature.

In order to verify the performance of MP-SIM, two kinds of specimens, such as step height sample with a discrete surface profile and a concave mirror with a continuously smooth surface, were measured. The step height sample was prepared with two gauge blocks (Grade 2, Mitutoyo) with 1.13 mm and 1.14 mm lengths, as shown in Figure 8a, and MP-SIM measured the surface profile of the boundary area of two gauge blocks. Figure 8b shows four phase-shifted illumination patterns on the surfaces of both gauge blocks as the current of the focus tunable lens increased. As shown in Figure 8b, the illumination pattern appeared at near the best focus of MP-SIM. Using Equation (5), the visibility of the pattern at each pixel of the PCMOS was calculated, and the 3D surface profile was reconstructed as shown in Figure 9a. As a result, the step height was calculated as 10.03 µm, corresponding to the height difference between two gauge blocks. For the repeatability, 10 consecutive measurements were performed, and the standard deviation of the measured step heights was calculated as 0.05 µm. The concave mirror with a 100 mm radius of curvature was also measured with the same optical configuration. Similar to the step height specimen, the surface profile was reconstructed as shown in Figure 9b. As a result of the radius of curvature calculation, it was 99.6 mm, similar to the specification of the manufacturer. The repeatability was 0.2 mm. 

In summary, the performance of the current MP-SIM has the measurement range of 570 µm, caused by current range of the focus tunable lens at the measurement area of (1.7 × 1.5 mm^2^). To evaluate the repeatability of measuring the surface profile, 10 consecutive measurement results of a plane mirror were obtained, and the mean value of the standard deviation for all pixels was calculated as 0.25 µm. Measurement time was determined as 5 s for the acquisition of 100 images by the exposure time and the frame rate of the PCMOS, because the response time of the TL was relatively shorter.

## 4. Discussion

**Figure 10:**
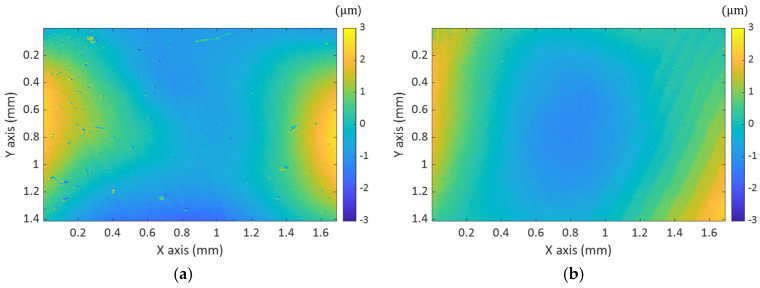
Distorted surface profile of a plane mirror caused by (**a**) astigmatism at the off-axis configuration and (**b**) spherical aberrations of the in-line configuration.

As the experimental results, the current version of MP-SIM has the capability of measuring the 3D surface profiles of various specimens without mechanical motions. However, MP-SIM needs to consider the aberration of the TL. In the current version of MP-SIM, the TL was put in front of the objective and it induced the aberrations. Even so, the optical axes of the lenses were not properly aligned, and the aberrations severely distorted the image, which led to the distortion of 3D surface profile. Figure 10a shows the 3D surface profile of a plane mirror reconstructed by MP-SIM when the optical axis of the TL is deviated from that of the system. 

As shown in Figure 10a, astigmatism affected the shape of the mirror, and the surface was seriously distorted. After the optical axes are in line, the astigmatism was reduced, but the measured surface was still curved, caused by the spherical aberration as shown in Figure 10b. To eliminate this aberration effect in this investigation, MP-SIM was calibrated with the measurement result of a plane mirror as accomplished in other techniques such as Moiré [31], and deflectometry [32]. Before measuring the specimen, a plane mirror was measured, and the result was subtracted from the measurement result of the specimen. Even though the optical design of the 4-f system further reduces the aberrations of the system, in this case, the system can be bulky, and the calibration with the measurement result of a plane mirror is more effective because it can reduce the systematic error, except for the aberrations. 

**Figure 11:**
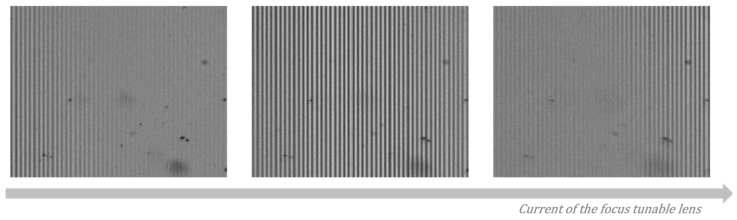
Localized illumination pattern and its movement by increasing the current of the TL.

MP-SIM uses polarizing structured illumination by using a polarization optic component, i.e., a Wollaston prism, instead of using a grid, as used in typical SIM, and the alignment of the WP becomes important for observing the pattern. In typical SIM, the illumination pattern is the result of imaging a grid, and the pattern can be obtained when the specimen is located at the best focus of the objective. Even though the grid or the specimen is slightly misaligned with a tilt, the pattern can be shown regardless of the coherence of the optical source. However, the illumination pattern of MP-SIM is generated by the interference between two divided beams, and the pattern can be localized when the low coherence source is used. Even so, the localization of the interference can be more severe when the WP is misaligned with a tilt. Figure 11 shows the polarizing illumination pattern when the WP is misaligned. 

**Figure 12:**
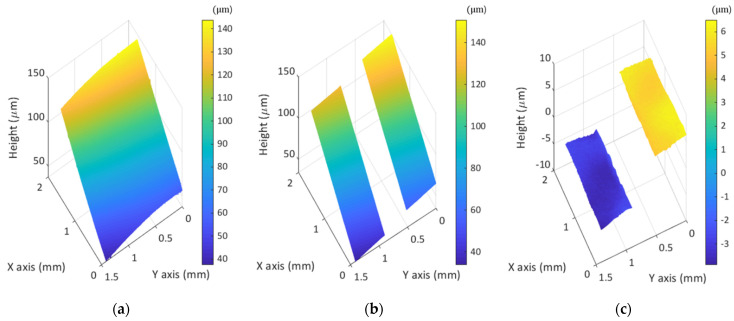
(**a**) inclined plane mirror result, (**b**) inclined step height sample result, and (**c**) calibrated result of a step height sample.

As shown in Figure 11, the interference pattern is severely localized, and the interference region is moved when the current of the TL increases. In this case, the measurement result of a plane mirror by MP-SIM is obtained as an inclined plane, as shown in Figure 12a. In addition, the reconstructed surface profile of a step height sample is also inclined as shown in Figure 12b. By calibrating the measurement result, which means the inclined plane mirror result is subtracted from the step height sample result, this misalignment effect is significantly mitigated, but the residual slope remains slightly, as shown in Figure 12c. Furthermore, the measurement error caused by the aberrations can be included in the measurement result as mentioned previously. In practicality, however, typical SIM has the same issues when the grid is misaligned with a tilt. As the illumination pattern is generated by imaging the grid pattern, the measurement result becomes inclined when the grid is misaligned similar to MP-SIM.

**Figure 13:**
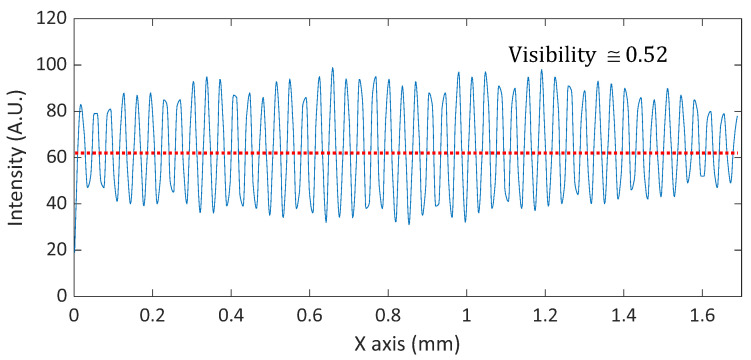
Illumination pattern and its visibility of MP-SIM.

One of the important issues in MP-SIM is lowering the visibility of the illumination pattern caused by the low coherence of the optical source. In this investigation, a band-pass filtered LED was used to eliminate the coherent noise, but two beams in MP-SIM experience different optical paths to each other, especially through the WP. Typically, a Wollaston prism is formed by two cemented triangular wedges of uniaxial birefringent material. The optical axis of one of the wedges is perpendicular to the plane of incidence that contains the normal to the interior interface, and the optical axis of the other wedge lies in this plane of incidence and is inclined relative to the entrance and the exit faces. Due to the structure of a Wollaston prism, optical path difference (OPD) between two beams occurs, and the visibility of the interference can be reduced [33]. In addition, the dispersion between two beams are not perfectly balanced. In order to prevent the reduction of the visibility, a thin Wollaston prism with a relatively small deviation angle was used in this investigation, and the visibility was calculated as 0.52 as shown in Figure 13. 

On the other hand, MP-SIM should sacrifice the lateral resolution because of the neighboring polarized pixels by a factor of two, and the number of whole available pixels are also reduced four times. This lateral resolution limit also restricts the spatial frequency of the interference pattern. Based on the Nyquist–Shannon sampling theory, the period of the interference pattern should be larger than twice the pixel size, and it should be larger than four times the pixel size of the PCMOS in MP-SIM. In the experiment, the period of the interference pattern was much larger than this limitation, and no image distortion occurred. However, it should be carefully considered when the higher magnification objective is used in the system because the interference fringe pattern becomes very dense.

The benefits of MP-SIM do not need the temporal phase-shifting to obtain the visibility of the pattern, which leads to minimizing the measurement time, and the visibility of the pattern can be obtained simultaneously. In typical SIM, three times the acquisition time of the image is required to apply the phase-shifting. Although CSSIM can reduce the measurement time by synchronizing the phase-shifting and the axial movement of the specimen, it needs more time to obtain the visibility of the pattern in post-processing because of the additional Fourier and inverse Fourier transformations. Another advantage of MP-SIM is that it does not have ghost illumination patterns, which can appear in typical SIM and CSSIM by the surface reflections of optical components because they use a low coherence light source, which can generate an interference pattern when the optical path difference is almost zero. Table 1 shows the comparison of typical SIM, CSSIM, and MP-SIM based on their fundamental measuring principles.

In the case of image acquisition and visibility calculation, the typical SIM needs more than three images in serial, but CSSIM and MP-SIM require a single shot image, even though MP-SIM obtains four images. Nowadays, the measurement speed of the system strongly depends on the image acquisition speed of the camera because the response time of a motorized stage, a DMD, an SLM, or a focus tunable lens is a few milliseconds, which can be negligible for the whole measurement time. When a CMOS camera with 60 fps is used in SIM, CSSIM, and MP-SIM, for example, SIM needs 50 ms for image acquisition, while it takes only 16.7 ms for CSSIM and MP-SIM. For the calculation time, typical SIM and MP-SIM do not need much time because the visibility can be obtained by simple arithmetic such as Equations (1) and (5). However, the visibility should be extracted by Fourier and inverse Fourier transformations in CSSIM. When those calculation times were compared to each other with the same calculation power, CSSIM needs more than 10 times the calculation time. The axial and lateral resolutions of typical SIM, CSSIM, and MP-SIM are dominantly affected by the numerical aperture (N.A.) of the objective [34]. In this case, they are in common for all systems based on the fundamental principles of SIM, as similar to CSM, if the spatial frequencies of the illumination patterns are the same. However, the lateral resolution of MP-SIM is worse, and the value is twice those of typical SIM and CSSIM because it uses a PCMOS, of which the unit cell is 2 × 2 pixels.

In MP-SIM, it is not easy to evaluate the measurement uncertainty of the system because there are too many parameters difficult to know and predict, such as the alignment of the Wollaston prism and speckles caused by the temporal coherence of the light source. However, the dominant uncertainty sources of MP-SIM can be considered, and they are phase-shifting error, intensity fluctuation of the light source, nonlinearity of the focus tunable lens, and optical aberrations caused by the practical misalignment of the optical components. In MP-SIM, the visibility is obtained by Equation (5) under the assumption of the same intensities for all polarization directions, as shown in Equation (4). If these intensities are different from each other, the visibility can be deviated from the exact value, which leads to measurement uncertainty. In this investigation, we used a linear polarizer in the optical source, which consists of a Glan–Thompson polarizer and a rotation mount, and adjusted the rotation of the transmission axis to balance the intensities for all directions in PCMOS. However, the remaining intensity imbalance and the extinction ratio of the polarizers deteriorate the visibility values. In addition, the temporal intensity fluctuation of the light source is also a critical factor in inducing measurement uncertainty. In MP-SIM, a focus tunable lens is used instead of axial scanning motion, and the linearity between the currents and the focal length shifts is very important. In this investigation, we calculate the conversion factor between the two, and estimate the nonlinearity as a peak deviation value from the linear curve. As a result, the nonlinearity was ±3 μm, which affects the repeatability of MP-SIM. As mentioned before, in spite of calibration by the measurement result of a plane mirror, the remaining aberration was also an uncertainty source of MP-SIM. 

## 5. Conclusions

In this investigation, we proposed and experimentally verified a motionless polarizing structured illumination microscopy based on the spatial phase-shifting technique to simultaneously obtain the visibility of the pattern. By using a Wollaston prism, the polarizing light pattern of two beams were generated, and four phase-shifted patterns were simultaneously captured by a polarization pixelated CMOS camera to calculate its visibility. Furthermore, an axial moving mechanism was replaced with a focus tunable lens. In the experimental result, a step height sample and a concave mirror were measured with 0.05 µm and 0.2 mm repeatabilities of step height and the radius of curvature, respectively.

## Figures and Tables

**Table 1:** Comparison of typical SIM, CSSIM and MP-SIM.

	Typical SIM	CSSIM	MP-SIM
Visibility calculation	Temporal phase-shifting	Fourier method (post-processing)	Spatial phase-shifting
Image acquisition	Three serial images	Synchronized single image	Four simultaneous images
Illumination pattern	Non-polarizing illumination pattern by grid, DMD, or SLM		Polarizing illumination pattern by a Wollaston prism
Axial resolution	Dependent on the numerical aperture of the objective		
Lateral resolution	Dependent on the numerical aperture of the objective		
Measurement speed *	fps of the camera × 3	fps of the camera	

* The response time of DMD, SLM, tunable lens, and motorized stage were not concerned.

## Data Availability

Not applicable.
